# Association between pre-stroke sarcopenia risk and stroke-associated infection in older people with acute ischemic stroke

**DOI:** 10.3389/fmed.2023.1090829

**Published:** 2023-02-23

**Authors:** Xiaodong Song, Xufeng Chen, Jie Bai, Jun Zhang

**Affiliations:** ^1^Department of Neurology, Peking University People’s Hospital, Beijing, China; ^2^Department of Neurology, Beijing Jishuitan Hospital, Beijing, China; ^3^Department of Infectious Diseases, The First Affiliated Hospital of Chongqing Medical University, Chongqing, China

**Keywords:** stroke-associated infection, pre-stroke sarcopenia risk, older people, acute ischemic stroke, SARC-F questionnaire

## Abstract

**Background:**

Stroke-associated infection (SAI) is a common complication after a stroke. The incidence of infection was higher in people with sarcopenia than in the general population. However, the relationship between pre-stroke sarcopenia risk and SAI in older patients has not been confirmed. This study aimed to investigate the association between pre-stroke sarcopenia risk and SAI in older patients with acute ischemic stroke (AIS).

**Methods:**

This retrospective study was conducted by the Peking University People’s Hospital. We evaluated the pre-stroke sarcopenia risk by applying the SARC-F questionnaire. Multivariate logistic regression was applied to explore the association between pre-stroke sarcopenia risk and SAI.

**Results:**

A total of 1,002 elder patients with AIS (592 men; 72.9 ± 8.6 years) were enrolled in our study. Pre-stroke sarcopenia risk was found in 29.1% of the cohort. The proportion of patients with pre-stroke sarcopenia risk was larger in the SAI group than in the non-SAI group (43.2 vs. 25.3%, *p*   < 0.001). In multivariate logistic analysis, pre-stroke sarcopenia risk was shown to be independently associated with SAI (OR = 1.454, 95% CI: 1.008–2.097, *p* = 0.045) after adjusting for potential factors. This association remained consistent across the subgroups based on age, sex, body mass index, smoking status, drinking status, diabetes, hypertension, and dyslipidemia.

**Conclusion:**

Pre-stroke sarcopenia risk was independently associated with SAI in older patients with AIS. Our findings highlight the significance of pre-stroke sarcopenia identification in the prevention and management of SAI in this population.

## Introduction

Stroke-associated infection (SAI) is a common complication after stroke (1). The rate of SAI was reported to be 30% (95% CI: 24–36%), with pneumonia and urinary tract infections (UTI) being the most common (2). SAI is associated with short-term mortality and adverse neurological functions (3, 4). It also has long-lasting negative effects on patients’ survival (5).

Sarcopenia was estimated in 15.8% of patients with acute stroke (6). Notably, pre-stroke sarcopenia is independently associated with severe disability and high mortality rates (7, 8). In addition, skeletal muscle has been considered an essential regulator of immune system function (9). Developing sarcopenia increased 3.88 times the risk of community-acquired pneumonia (10). Moreover, the risk of infection was increased by 49.6% [odds ratio (OR) = 1.496, 95% confidence interval (CI): 1.102–2.031] in patients with diabetes and sarcopenia than in those without (11). Therefore, it is necessary to pay attention to the risk of SAI in patients with sarcopenia and acute ischemic stroke (AIS).

Muscle mass and quality evaluation occupy an important position in diagnosing sarcopenia (12). However, acute stroke often led to a sudden disorder of physical function and consciousness. The diagnosis of sarcopenia is difficult to be performed in such a clinical setting. SARC-F is a brief questionnaire for sarcopenia screening and frees researchers from measuring muscle function (13–15). Therefore, we aimed to determine whether the sarcopenia risk defined by SARC-F is a risk factor for SAI in older patients after AIS.

## Methods

### Patient selection

We retrospectively enrolled the older patients suffering from AIS in the Peking University People’s Hospital from September 2019 to February 2022. The inclusion criteria were as follows: age ≥ 60 years, admission within 24 h of symptom onset, and a neurological deficit symptom with infarction lesion on imaging. The exclusion criteria included the following: (1) intracranial hemorrhage or subarachnoid hemorrhage; (2) cannot complete the SARC-F questionnaire due to dementia, aphasia, and loss of consciousness; (3) active infection within the last 2 weeks; (4) premorbid stroke-related disability; (5) active malignancy; and (6) recent history of trauma or surgery.

### Data collection

Data were abstained at the time of presentation, including age, sex, body mass index (BMI), smoking status, drinking status, stroke etiology, National Institutes of Health Stroke Scale (NIHSS) score, diabetes, hypertension, dyslipidemia, hyperuricemia, coronary artery disease, chronic heart failure, and chronic obstructive pulmonary disease. According to the Trial of Org 10,172 in Acute Stroke Treatment classification, AIS was categorized into five etiologies: large-artery atherosclerosis, cardioembolism, small vessel occlusion, other determined etiologies, and undetermined etiology (16). Blood samples were taken within 24 h of admission. Laboratory data were collected, including hemoglobin (HB), fast blood glucose, serum creatinine, estimated glomerular filtration rate (eGFR), uric acid, total bilirubin, direct bilirubin, serum album (ALB), alanine aminotransferase, total cholesterol, total glyceride, and D-dimmer.

### Pre-stroke sarcopenia risk assessment

To evaluate pre-stroke sarcopenia risk, patients were asked to complete the SARC-F questionnaire by recalling within 2 days of admission. SARC-F is a simple questionnaire for rapidly identifying people at risk of sarcopenia (13). It has five items: strength, assistance in walking, rising from a chair, climbing stairs, and falls. Each item will be assigned a score of 0–2 (15), depending on the individual physical performance before stroke onset. The SARC-F score range is 0 to 10. Patients were categorized as having pre-stroke sarcopenia risk if the SARC-F score ≥ 4 (15).

### Outcome definition

Stroke-associated infection was defined as any new infection within the first week of AIS onset (17, 18). Patients diagnosed with the infection must meet the modified Centers for Disease Control and Prevention criteria (19). SAI was classified into three types, including pneumonia, UTI, and other infection. Pneumonia was suspected when there were relevant clinical symptoms and leukocytosis (>11 × 10^9^/L) and confirmed with an infiltrate on the chest radiograph. UTI was diagnosed according to the urinary tract symptoms and positive microbiological cultures (using midstream urine). Other infection diagnoses were made based on their corresponding diagnostic criteria.

### Statistical analysis

Statistical analyses were conducted using IBM SPSS Statistics version 22.0 (IBM Corp), R project for Statistical Computer version 4.1.2,^1^ and MedCalc Statistical Software version 20.1.0 (MedCalc Software Ltd). A *p*-value of <0.05 was defined as statistically significant. The differences between the two groups in continuous variables were evaluated by the *t*-test or Mann–Whitney *U* test. The comparisons of categorical variables were performed using Fisher’s exact test and Pearson’s chi-square test. Univariate logistic regression analysis was used to screen for the potential factors associated with SAI. Then, the factors with a value of *p* of <0.05 were added to stepwise multivariate logistic regression to explore the independent factors of SAI.

## Results

### Patient characteristics

**Figure 1:**
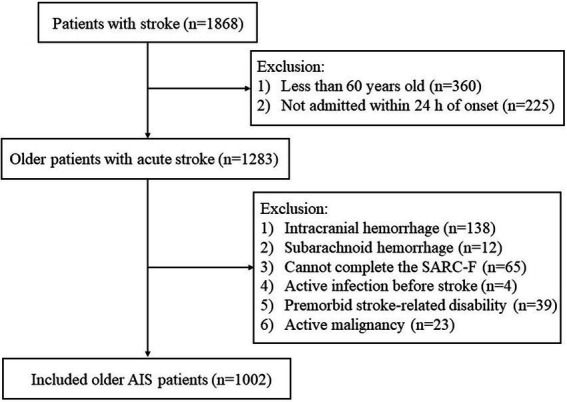
Flow diagram of patients’ selection. AIS, acute ischemic stroke.

A total of 1,002 patients were enrolled in our research cohort after applying the selection criteria (Figure 1). There were 215 (21.5%) patients suffering from SAI during hospitalization, 104 of which were diagnosed with pneumonia, 66 with UTI, 42 with other infections, and 3 with both pneumonia and UTI. Patient’s characteristics classified by pre-stroke sarcopenia risk are shown in Table 1. The sarcopenia risk group was older (*p* < 0.001) and had a higher NIHSS score (*p* < 0.001) than its counterpart. Patients with sarcopenia risk had a higher level of serum creatinine and lower levels of HB, eGFR, and ALB than those without (all *p* ≤ 0.05). In addition, the proportion of coronary artery disease was bigger in the sarcopenia risk group (*p* = 0.005).

**Table 1:** Demographic and clinical variables of patients.

Variable	All cases (*n* = 1,002)	Without sarcopenia risk (*n* = 710)	With sarcopenia risk (*n* = 292)	*p*-value
Age (years), median (IQR)	72.0 (66.0–80.0)	70.0 (65.0–78.0)	77.0 (69.0–83.0)	**<0.001** ^*^
Male, n (%)	592 (59.1)	407 (57.3)	185 (63.3)	0.078
Smoking, n (%)	328 (32.7)	240 (33.8)	88 (30.1)	0.261
Drinking, n (%)	222 (22.2)	160 (22.5)	62 (21.2)	0.652
BMI (kg/m^2^), mean (SD)	23.2 (4.6)	23.4 (4.7)	22.8 (4.4)	0.058
Stroke etiology				0.526
Large-artery atherosclerosis	546 (54.5)	390 (54.9)	156 (53.4)	
Cardioembolism	78 (7.9)	50 (7.0)	28 (9.6)	
Small vessel occlusion	202 (20.2)	149 (21.0)	53 (18.2)	
Other determined etiology	57 (5.7)	38 (5.4)	19 (6.5)	
Undetermined etiology	119 (11.9)	83 (11.7)	36 (12.3)	
NIHSS, median (IQR)	3.0 (2.0–6.0)	3.0 (2.0–5.0)	5.0 (3.0–8.0)	**<0.001** ^*^
Diabetes, n (%)	393 (39.2)	267 (37.6)	126 (43.2)	0.102
Hypertension, n (%)	757 (75.5)	533 (75.1)	224 (76.7)	0.583
Dyslipidemia, n (%)	662 (66.1)	460 (64.8)	202 (69.2)	0.233
Coronary artery disease, n (%)	240 (24.0)	153 (21.5)	87 (29.8)	**0.005**
Chronic heart failure, n (%)	13 (1.3)	9 (1.3)	4 (1.4)	1
Atrial fibrillation, n (%)	78 (7.8)	50 (7.0)	28 (9.6)	0.172
COPD, n (%)	35 (3.5)	26 (3.7)	9 (3.1)	0.65
HB (g/L), median (IQR)	134.0 (123.0–144.0)	135.0 (125.0–145.0)	132.0 (120.0–142.0)	**0.004**
FBG (mmol/L), median (IQR)	7.1 (5.9–9.3)	7.0 (5.9–9.2)	7.3 (6.0–9.6)	0.147
SCr (μmol/L), median (IQR)	70.0 (58.0–87.0)	69.0 (58.0–85.0)	73.0 (60.8–94.0)	**0.008**
eGFR (mL/min*1.73m^2^), median (IQR)	85.0 (68.0–93.8)	87.2 (71.0–94.7)	80.4 (61.5–90.7)	**<0.001** ^*^
Uric acid (μmol/L), median (IQR)	315.0 (252.3–386.0)	315.5 (256.0–386.8)	315.0 (242.8–378.3)	0.65
Total bilirubin (μmol/L), median (IQR)	12.2 (9.6–16.0)	12.4 (9.7–16.0)	12.0 (9.3–16.1)	0.427
Direct bilirubin (μmol/L), median (IQR)	4.0 (3.1–5.4)	4.1 (3.2–5.5)	4.0 (3.0–5.2)	0.27
ALT (U/L), median (IQR)	16.0 (12.0–24.0)	16.0 (12.0–24.0)	16.0 (12.0–23.0)	0.977
ALB (g/L), mean (SD)	37.7 (4.3)	38.0 (4.2)	36.9 (4.5)	**0.001**
TC (mmol/L), median (IQR)	4.2 (3.4–5.0)	4.2 (3.4–5.0)	4.3 (3.5–5.1)	0.199
TG (mmol/L), median (IQR)	1.4 (1.1–2.0)	1.4 (1.1–1.9)	1.5 (1.1–2.0)	0.418
D-dimmer (μmol/L), median (IQR)	145.0 (82.0–323.0)	147.0 (80.0–332.0)	142.5 (86.0–291.0)	0.801

SD, standard deviation; IQR, interquartile range; BMI, body mass index; NIHSS, National Institutes of Health Stroke Scale; COPD, chronic obstructive pulmonary disease; HB, hemoglobin; FBG, fast blood glucose; SCr, serum creatinine clearance; eGFR, estimated glomerular filtration rate; ALT, alanine aminotransferase; ALB, album; TC, total cholesterol; TG, triglyceride. Bold means value of *p* of ≤ 0.05; Bold^*^ means value of *p* of ≤ 0.001.

### Association between pre-stroke sarcopenia and SAI

**Figure 2:**
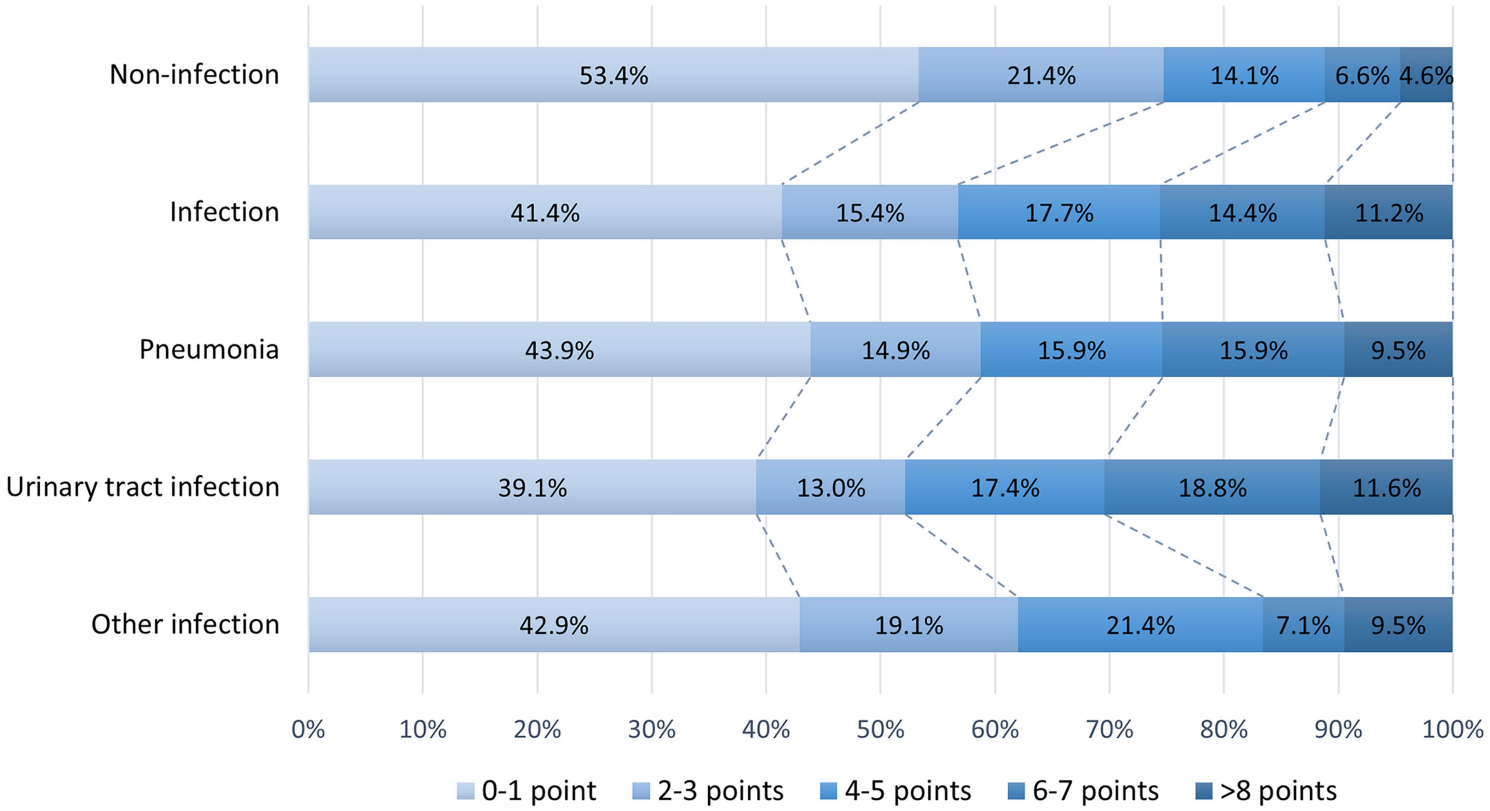
Distribution of SARC-F scores in patients with and without SAI. SAI, stroke-associated infection.

Figure 2 shows the distribution of the SARC-F score before stroke onset in patients. Pre-stroke sarcopenia risk identified by the SARC-F questionnaire (≥4 points) was found in 29.2% of older patients with AIS. The proportion of patients with pre-stroke sarcopenia risk was larger in the SAI group than in the non-SAI group (43.2 vs. 25.3%, *p* < 0.001). Similarly, the ratios of pre-stroke sarcopenia risk were higher in both patients with pneumonia (41.1 vs. 25.3%) and UTI (47.8 vs. 25.3%) than those without SAI (all *p* ≤ 0.05). Patients with other infections had a higher rate of sarcopenia risk compared with those without infection (38.1 vs. 25.3%), though the difference was not significant (*p* = 0.065).

**Figure 3:**
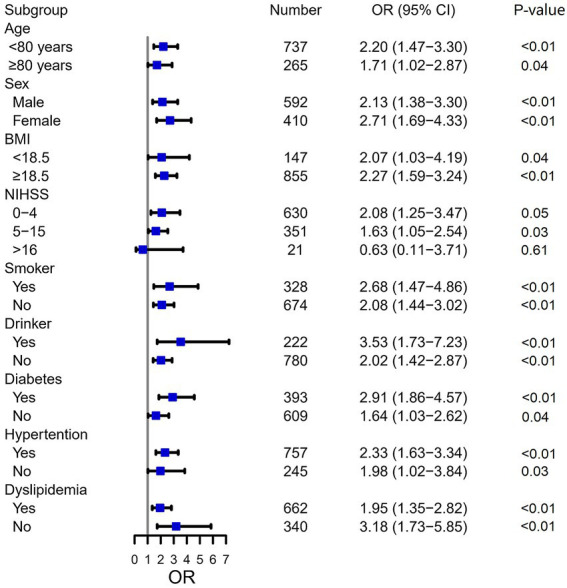
Subgroup analysis of sarcopenia risk for SAI. SAI, stroke-associated pneumonia; OR, odds ratio; CI, confidence interval; BMI, body mass index; NIHSS, National Institutes of Health Stroke Scale.

In univariate logistic regression analysis, the OR of SAI was 2.252 (95% CI, 1.645–3.084, *p* < 0.001) for patients with pre-stroke sarcopenia risk. The association between pre-stroke sarcopenia risk and SAI persisted (OR = 1.454, 95% CI: 1.008–2.097, *p* ≤0.05) after adjustment for age, BMI, NIHSS score at admission, diabetes, and ALB (Table 2). Subgroup analyses were performed to investigate whether the effect of sarcopenia risk on SAI occurrence was consistent among different patients (Figure 3). Pre-stroke sarcopenia risk did not increase the risk of SAI in patients with NIHSS score > 16 (*p* = 0.605). However, pre-stroke sarcopenia risk was significantly associated with SAI among diverse groups categorized by age, sex, BMI, smoking status, drinking status, diabetes, hypertension, and dyslipidemia (all *p* < 0.05) (Figure 3).

**Table 2:** Univariate and multivariate regression analysis of SAI.

Variable	Univariate regression		Multivariate regression	
	OR (95% CI)	*p*-value	OR (95% CI)	*p*-value
Age (years)	1.054 (1.035–1.073)	**<0.001**	1.028 (1.007–1.049)	**0.008**
Male	0.560 (0.414–0.760)	**<0.001**		
Smoking	0.667 (0.476–0.935)	**0.019**		
Drinking	0.731 (0.498–1.074)	0.111		
BMI (kg/m^2^)	0.886 (0.851–0.922)	**<0.001**	0.911 (0.872–0.952)	**<0.001** ^*^
Sarcopenia risk	2.252 (1.645–3.084)	**<0.001**	1.454 (1.008–2.097)	**0.045**
Stroke etiology				
Large-artery atherosclerosis	Reference			
Cardioembolism	1.567 (0.924–2.658)	0.096		
Small vessel occlusion	0.925 (0.619–1.384)	0.706		
Other determined etiology	0.797 (0.391–1.627)	0.534		
Undetermined etiology	1.100 (0.684–1.770)	0.695		
NIHSS categories				
0–4	Reference		Reference	
5–15	3.663 (2.656–5.051)	**<0.001**	2.624 (1.842–3.738)	**<0.001** ^*^
>16	11.172 (4.491–27.794)	**<0.001**	5.801 (2.047–16.44)	**0.001**
Diabetes	2.437 (1.793–3.312)	**<0.001**	1.962 (1.391–2.769)	**<0.001** ^*^
Hypertension	1.162 (0.811–1.664)	0.414		
Dyslipidemia	1.809 (1.282–2.553)	**0.001**		
Coronary artery disease	0.861 (0.600–1.237)	0.418		
Chronic heart failure	2.319 (0.751–7.161)	0.144		
Atrial fibrillation	1.594 (0.956–2.660)	0.074		
COPD	1.964 (0.961–4.134)	0.064		
HB (g/L)	0.991 (0.982–1.000)	0.058		
FBG (mmol/L)	1.046 (1.010–1.082)	**0.010**		
SCr (μmol/L)	1.000 (0.998–1.002)	0.804		
eGFR (ml/min*1.73 m^2^)	0.992 (0.985–0.998)	**0.013**		
Uric acid (μmol/L)	1.000 (0.999–1.002)	0.785		
Total bilirubin (μmol/L)	1.019 (0.998–1.040)	0.083		
Direct bilirubin (μmol/L)	0.955 (0.897–1.016)	0.147		
ALT (U/L)	0.990 (0.979–1.001)	0.070		
ALB (g/L)	0.830 (0.799–0.863)	**<0.001**	0.866 (0.831–0.901)	**<0.001** ^*^
TC (mmol/L)	0.971 (0.864–1.091)	0.624		
TG (mmol/L)	1.009 (0.909–1.121)	0.861		
D-dimmer (μmol/L)	0.999 (0.997–1.001)	0.359		

OR, odds ratio; CI, confidence interval; BMI, body mass index; NIHSS, National Institutes of Health Stroke Scale; COPD, chronic obstructive pulmonary disease; HB, hemoglobin; FBG, fast blood glucose; SCr, serum creatinine clearance; eGFR, estimated glomerular filtration rate; ALT, alanine aminotransferase; ALB, album; TC, total cholesterol; TG, triglyceride. Bold means a value of *p* of ≤ 0.05; Bold^*^ means a *p*-value of ≤ 0.001.

## Discussion

Our research revealed that pre-stroke sarcopenia risk was independently associated with SAI in older patients with AIS. Moreover, the association remained significant in diverse subgroups categorized by clinical characteristics. These results indicate the significance of pre-stroke sarcopenia identification in the prevention and management of SAI in elders.

In Chinese hospitalized elder adults, the prevalence of sarcopenia for men and women was 29.7% (95% CI 18.4–41.1%) and 23% (95% CI 17.1–28.8%), respectively (20). Consistently, pre-stroke sarcopenia risk was found in 29.2% of older patients with AIS in the present study. Sarcopenia is an age-related process occurring in older adults (21). Hence, it is not surprising that patients with pre-stroke sarcopenia risk were older than those without sarcopenia risk. Additionally, patients with sarcopenia risk had lower levels of ALB and HB compared with their counterparts in our study. The low level of ALB and HB tends to suggest malnutrition, which is an independent risk factor for sarcopenia in the elderly (22).

Age, NIHSS score, diabetes, and ALB were confirmed to be associated with SAI in the previous research (17, 23). After adjusting for these potential factors, pre-stroke sarcopenia risk was still an independent predictor (OR = 1.454, 95% CI: 1.008–2.097) for SAI in the present study. In line with our results, sarcopenia was shown to be an essential predictor for postoperative infections (24–26). In addition, sarcopenia doubles the risk of nosocomial infection in elder people admitted to acute care after 3 weeks of hospitalization (27). Skeletal muscle is increasingly accepted as a regulator of the immune system (9). It can modulate immune functions by secreting myokines, such as IL-7 and IL-15 (9). IL-7 is considered an essential signal for the survival and expansion of mature T cells (28). IL-15 plays a key role in the proliferation, activation, and distribution of natural killer cells and CD8 T cells (29). Moreover, stroke can result in an impairment of the defense mechanisms (1). Thus, we presumed that sarcopenia might aggravate the impaired immune response to pathogens in older patients with AIS.

Apart from impaired immune function, the decline in muscle quantity and quality might be another non-negligible cause of SAI. The tongue muscle is considered an essential swallowing-related muscle. Sarcopenia is associated with swallowing disorder in older adults (30, 31), partly due to a decrease in the mass of tongue muscles (32). Moreover, swallowing dysfunction increased the incidence of aspiration in the elderly, leading to airway colonization of Gram-negative bacteria (33). Thus, sarcopenia may contribute to pneumonia in patients with AIS through the same pathogenesis. Besides pneumonia, UTI (6.9%) was the second most common infection in our cohort. Urinary incontinence rate was elevated in older women with sarcopenia, with the declining function of pelvic muscles being the possible reason (34). Notably, urinary incontinence may increase the risk of UTI in the older population (35, 36). Hence, patients with sarcopenia risk were more likely to have UTI in our study.

There are some limitations to our study. First, we eliminated the patients with aphasia, dementia, and consciousness disorder since they could not complete a questionnaire. The selected bias may limit the generalizability of our findings. Second, the pre-sarcopenia risk was diagnosed based on the SARC-F questionnaire. It was considered a screening tool with low sensitivity but high specificity (15). For this reason, the number of sarcopenia in our cohort may be underestimated. Third, this is a single-center retrospective study with a limited sample size. Multicenter prospective studies are expected to validate the association between pre-stroke sarcopenia risk and SAI in older patients with AIS.

## Conclusion

Pre-stroke sarcopenia risk was independently associated with SAI in older patients with AIS. Our findings highlight the significance of pre-stroke sarcopenia identification in the prevention and management of SAI in older people.

## Data availability statement

The original contributions presented in the study are included in the article/supplementary material, further inquiries can be directed to the corresponding authors.

## Ethics statement

The studies involving human participants were reviewed and approved by the Ethics Committee of Peking University People’s Hospital. Written informed consent for participation was not required for this study in accordance with the national legislation and the institutional requirements.

## Author contributions

JB was involved in the conception and design of the study. JZ contributed to the data analysis and interpretation. XS and XC collected the clinical data and wrote the manuscript. All authors discussed and approved the final manuscript.

## Conflict of interest

The authors declare that the research was conducted in the absence of any commercial or financial relationships that could be construed as a potential conflict of interest.

## Publisher’s note

All claims expressed in this article are solely those of the authors and do not necessarily represent those of their affiliated organizations, or those of the publisher, the editors and the reviewers. Any product that may be evaluated in this article, or claim that may be made by its manufacturer, is not guaranteed or endorsed by the publisher.

## Footnotes

^1^ http://www.r-project.org/
